# Acute management and outcome of multiple trauma patients with pelvic disruptions

**DOI:** 10.1186/cc11487

**Published:** 2012-08-22

**Authors:** Markus Burkhardt, Ulrike Nienaber, Antonius Pizanis, Marc Maegele, Ulf Culemann, Bertil Bouillon, Sascha Flohé, Tim Pohlemann, Thomas Paffrath

**Affiliations:** 1 Department of Trauma, Hand and Reconstructive Surgery, University of Saarland, Kirrbergerstr. 100, 66421 Homburg/Saar, Germany; 2 AUC-Academy of Trauma Surgery, Landwehrstr. 34, 80336 Munich, Germany; 3 Department of Trauma and Orthopedic Surgery, University of Witten/Herdecke, Cologne-Merheim Medical Centre (CMMC), Ostmerheimerstr. 200, 51109 Cologne, Germany; 4 Department of Trauma and Hand Surgery, University Hospital Düsseldorf, Moorenstrasse 5, 40225 Düsseldorf, Germany

## Abstract

**Introduction:**

Data on prehospital and trauma-room fluid management of multiple trauma patients with pelvic disruptions are rarely reported. Present trauma algorithms recommend early hemorrhage control and massive fluid resuscitation. By matching the German Pelvic Injury Register (PIR) with the TraumaRegister DGU (TR) for the first time, we attempt to assess the initial fluid management for different *Tile/OTA *types of pelvic-ring fractures. Special attention was given to the patient's posttraumatic course, particularly intensive care unit (ICU) data and patient outcome.

**Methods:**

A specific match code was applied to identify certain patients with pelvic disruptions from both PIR and TR anonymous trauma databases, admitted between 2004 and 2009. From the resulting intersection set, a retrospective analysis was done of prehospital and trauma-room data, length of ICU stay, days of ventilation, incidence of multiple organ dysfunction syndrome (MODS), sepsis, and mortality.

**Results:**

In total, 402 patients were identified. Mean ISS was 25.9 points, and the mean of patients with ISS ≥16 was 85.6%. The fracture distribution was as follows: 19.7% type A, 29.4% type B, 36.6% type C, and 14.3% isolated acetabular and/or sacrum fractures. The type B/C, compared with type A fractures, were related to constantly worse vital signs that necessitated a higher volume of fluid and blood administration in the prehospital and/or the trauma-room setting. This group of B/C fractures were also related to a significantly higher presence of concomitant injuries and related to increased ISS. This was related to increased ventilation and ICU stay, increased rate of MODS, sepsis, and increased rate of mortality, at least for the type C fractures. Approximately 80% of the dead had sustained type B/C fractures.

**Conclusions:**

The present study confirms the actuality of traditional trauma algorithms with initial massive fluid resuscitation in the recent therapy of multiple trauma patients with pelvic disruptions. Low-volume resuscitation seems not yet to be accepted in practice in managing this special patient entity. Mechanically unstable pelvic-ring fractures type B/C (according to the Tile/OTA classification) form a distinct entity that must be considered notably in future trauma algorithms.

## Introduction

**Figure 1:**
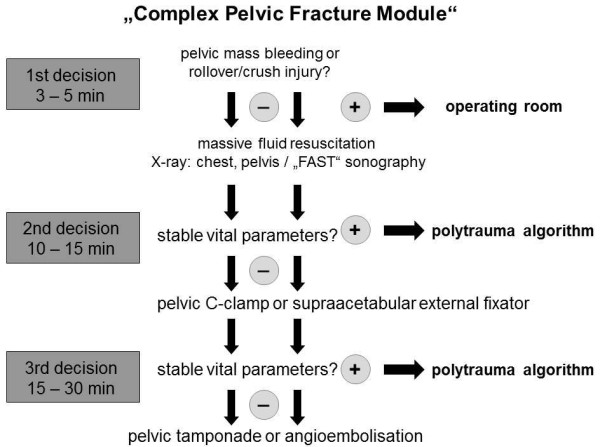
**Modified "Complex Pelvic Fracture Module" according to Tscherne and Pohlemann as part of the trauma algorithm in the emergency department or trauma room **[14]. (With kind permission of Springer Science+Business Media).

Disruptions of the pelvic ring represent 2% to 3% of all fractures. The injury mechanisms most likely to cause a pelvic fracture are high-speed road traffic accidents and falls from high altitude. In multiple-trauma patients, the incidence of pelvic injuries is increased, and pelvic hemorrhage may result from bony bleeding or disruptions of the surrounding perivesical or presacral venous plexus, but also arterial pelvic vessels have been implicated as a significant factor leading to exsanguination and death [1-3]. The reported mortality rates differ largely from 5% to 50% and are dependent not only on the type of pelvic-ring fracture but also on the severity of associated injuries involving the abdomen, chest, and central nervous system [3-10]. Although, according to recent literature, a distinct decrease of mortality rate is found in patients with a combination of severe pelvic disruptions and hemodynamic instability or with so-called "complex pelvic injuries," it still remains unacceptably high [10,11]. Complex pelvic injuries are defined as all pelvic fractures (acetabulum, pelvic ring, and sacrum) with pelvic soft-tissue injuries (that is, open fracture including Morel-Lavallée lesion, disruption of pelvic vessels including retroperitoneal hematoma, and urogenital and neurologic injuries directly related to the pelvic fracture) [1]. For these seriously injured patients, a multitude of scientific investigations and as many management guidelines exist [12-20]. Most of the latter focus on the patient's time in the emergency department (ED) or trauma room, and examples of actual guidelines are the "Complex Pelvic Fracture Module," as part of the trauma algorithm by Tscherne and Pohlemann (Figure 1) and the ATLS Pelvic Fracture Algorithm (Advanced Trauma Life Support). Both pelvis-specific trauma algorithms recommend early hemorrhage control and massive fluid resuscitation [13,17]. Thereby, hemorrhage control is achieved through mechanical immobilization of the pelvic ring by external counterpressure with the aid of a pelvic binder, a pelvic C-clamp, or an external fixator. In certain cases, ongoing pelvic hemorrhage may require pelvic packing and/or angioembolization. In contrast to the emergency department or trauma room well-established management guidelines, data on the prehospital management of pelvic fractures and its relation to outcome is rarely reported in the literature. This is rather regrettable because of the upcoming evidence that limiting the amount of fluids given by following a strategy of permissive hypotension during the initial resuscitation period may improve trauma outcomes [21-23]. Although the German Pelvic Injury Register (PIR) represents the only nationwide database specifically focusing on pelvic trauma, unfortunately, a scarcity exists of records of the prehospital phase, and the trauma room and intensive care unit (ICU) data are fewer. Conversely, the TraumaRegister DGU (TR) includes all these missing data, but all included injuries, even the pelvic fractures, are coded by using the Abbreviated Injury Scale (AIS). A further difficulty is appearing from the fact that, in contrast to the Tile/OTA (Orthopaedic Trauma Association) classification that is used in the PIR, the AIS is not adopted in recent trauma algorithms nor is it in clinical use [24]. We report herein for the first time on matching these two anonymous trauma databases to create an intersection set that benefits from complementary data of each register, respectively. We focus on not-yet-described differences in fluid management for different *Tile/OTA *types of pelvic-ring fractures in the initial resuscitation period (that is, prehospital phase and time from arrival in the trauma room until admission to the ICU). We would also like to find out whether, apart from the "complex pelvic fractures," the mechanically unstable pelvic-ring fractures type B/C, according *Tile's/OTA *classification, form a distinct entity that must be considered separately within future pelvic-trauma algorithms.

## Materials and methods

### The German Pelvic Injury Register

The PIR represents the only nationwide database specifically focusing on pelvic trauma and contains detailed information on demographics, fracture classification, in-hospital management with the main focus on timing and manner of operative treatment, relevant laboratory findings including data on transfusion, and outcome of at least each operative treatment. The register is approved by the review board of the German Society for Trauma Surgery and is in compliance with institutional requirements. All pelvic fractures were classified by experienced orthopedic surgeons by using the Tile classification adopted by the OTA [24]. Mechanically stable pelvic-ring fractures were classified as A type; fractures with rotational instability alone, as B type; and fractures with both rotational and translational instability, as C-type injuries. Classifications were based on plain radiographs and computed tomography scans. As the PIR is an anonymous register, the Institutional Review Board waived the need for patient consent.

### The TraumaRegister DGU

The TR is a prospective, multicenter, standardized, and anonymous documentation of multiply injured trauma patients at four consecutive posttrauma phases from injury to hospital discharge: (a) prehospital phase; (b) trauma room and initial surgery (until admission to ICU); (c) ICU; and (d) outcome status at discharge when description of injuries and procedures takes place. The register contains detailed information on demographics, injury pattern, comorbidities, pre- and in-hospital management, time course, relevant laboratory findings including data on transfusion, and outcome of each individual. The TR is a voluntary register approved by the review board of the German Society for Trauma Surgery and is in compliance with institutional requirements. In accordance to the PIR, the TR is a voluntary and anonymous register that needs no patient consent.

### Data collection and match code

We started with the evaluation of the smaller PIR that included from 2004 to 2009 a total number of 4,323 patients collected by at least 31 participating trauma departments. Notably, only 19 hospitals concomitantly shared their data with the TR, so at least 3,329 anonymous patients of the PIR potentially registered their data in both databases. Looking at the TR, the initial 34,134 trauma victims from 242 affiliated hospitals during the same observation period were reduced to the same PIR-affiliated hospitals and furthermore screened for the AIS code 856xxx.x, reflecting pelvic-ring and acetabular fractures, thus resulting in 1,974 trauma victims with pelvic fractures. Because of the uneven yearly hospital contribution to the registers, in the next step, we focused on the time overlapping contribution to both registers, resulting in further decreased raw data of potentially twice-documented patients (that is, 2,671 patients from the PIR and 947 patients from the TR). These patients were matched by using a specific match code for both registers, including the code of the trauma department, date of admission, date of discharge, age, and sex of the patient. After exclusion of 10 duplicates, data records of both registers of in all 420 patients from 15 hospitals were finally linked together. The corresponding match rates for the intersection set were 15.7% in the PIR and 44.4% in the TR. In accordance with Sathy *et al*. [25], patients with an unfavorable prognosis such as AIS head >4 (*n *= 18) were excluded before a retrospective analysis was performed.

### Used definitions for initial resuscitation period, multiple organ dysfunction syndrome (MODS), and sepsis

The Initial Resuscitation Period was defined as the time interval from the beginning of the prehospital phase until admission to the ICU after being treated in the ED or trauma room. Notably, the precise length of this time interval is not documented in the PIR or the TR, but usually is less than 24 hours. In the TR, the MODS is assessed by using the Sequential Organ Failure Assessment (SOFA) score [26,27]. Three of four SOFA points are considered to have organ dysfunction. For each organ or function (that is, lung, liver, kidney, coagulation system, cardiovascular system, and central nervous system), the number of days with organ dysfunction in the ICU is recorded. In addition, the number of days with MODS (at least two organs with dysfunction) is recorded. The incidence of sepsis was assessed according to Bone *et al*. [28].

### Statistical analysis

Demographic and clinical data are presented as mean ± standard deviation (SD) and as percentages for categoric variables. For continuous variables, normal distribution was analyzed with the Shapiro-Wilk test and showed all data as not normally distributed. Patients were subdivided into three groups depending on the type of pelvic-ring fracture (type A, B, or C). Complex pelvic injuries were also investigated. Isolated acetabular and/or sacrum fractures were excluded, as we considered these injuries not to have a substantial impact on acute management or hospital mortality [10]. Regarding mortality, we further compared complex with noncomplex pelvic injuries and analyzed the fracture distribution, even in the nonsurviving group. To detect differences between these patient groups, a Kruskal-Wallis test was performed. In case of a significant overall difference, pairwise comparison was performed with a Mann-Whitney *U *test. Categoric variables were analyzed accordingly with a χ^2 ^test. Statistics were calculated by using SPSS Statistical Software Package Version 19 (SPSS, Inc., Chicago, IL, USA). A *P *value of <0.05 was considered statistically significant.

## Results

**Figure 2:**
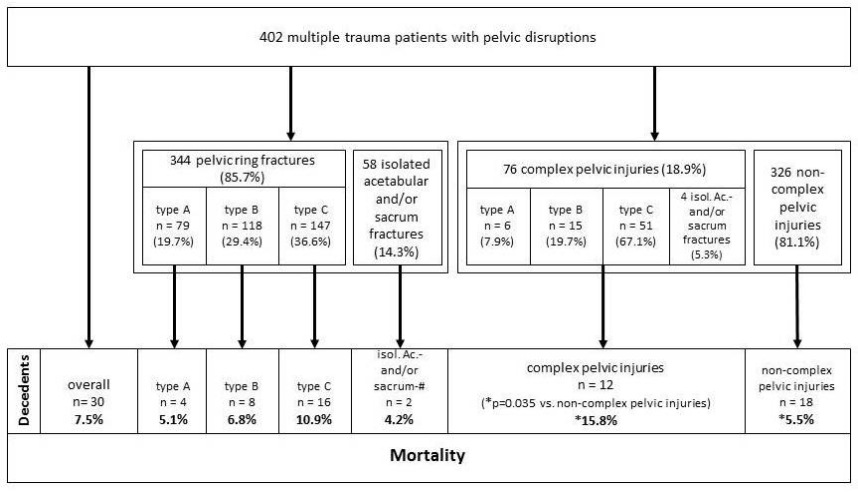
**Mortality of multiple-trauma patients with pelvic disruptions**. Pelvic-ring fractures were classified according to the Tile/OTA classification [24]. Pelvic fractures were classified as complex pelvic injuries whenever they were associated with a significant organ or soft-tissue damage [1].

After exclusion of 18 patients with unfavorable prognosis due to severe traumatic brain injury (AIS head >4), 402 multiple-trauma patients with pelvic disruptions were under investigation. The distribution of the pelvic disruptions revealed, in the majority, pelvic-ring fractures divided into 19.7% type A (*n *= 79), 29.4% type B (*n *= 118), and 36.6% type C (*n *= 147). In addition, 14.3% (*n *= 58) isolated acetabular fractures and/or fractures of the sacrum were found. Table 1 gives the main characteristics of the investigated patients. No significant differences were noted between the fracture groups in the ratio of blunt injury, age, gender, GCS on scene, time from accident to hospital admission, and time in the trauma room. In contrast, the ISS, the ratio of ISS ≥16, as well as the New-ISS and PTS, revealed significantly more severely injured patients with pelvic-ring fractures types B/C compared with type A. On AIS breakdown, more severe (AIS ≥3) head injuries were related to types A and B. Conversely, thoracic trauma did not have predominance in any of the three types, but types B and C were related to more abdominal and extremity injuries. Complex pelvic injuries were identified in 18.9% (*n *= 76), and the distribution showed a distinct shift to the rotationally and/or translationally unstable pelvic-ring fractures types B/C (that is, 7.9% type A (*n *= 6), 19.7% type B (*n *= 15), 67.1% type C (*n *= 51), and 5.3% isolated acetabular fractures and/or fractures of the sacrum (*n *= 4). With increasing pelvic-ring instability, the incidence of complex pelvic injuries increased to 7.6% for type A, 12.7% for type B, and 34.7% for type C. The overall mortality was 7.5% (*n *= 30). Of the pelvic-ring subgroups, type C fractures showed the highest mortality, but this was not statistically significant. In the non-complex-injury group, a 5.5% mortality rate was found compared with 15.8% of the patients with complex pelvic injuries (Figure 2). Of the 30 patients who died, 53% had sustained type C; 27.7%, type B; 13.3%, type A; and 6.7%, isolated acetabular and/or sacral fractures. Therefore, approximately 80% of the patients who died sustained a type B/C pelvic ring fracture.

**Table 1:** Basic characteristics of the 402 trauma patients with pelvic fracture and the 344 pelvic-ring fractures were classified according to Tile/OTA classification

		Type of pelvic-ring fracture according Tile/OTA fracture classification				Pair-wise comparison		
	
	Total	Type A	Type B	Type C	Overall test	*P *value (A vs. C)	*P *value (B vs. C)	*P *value (A vs. B)
Blunt injury (%)	97.9 (*n *= 393)	97.4 (*n *= 76)	99.1 (*n *= 116)	99.3 (*n *= 145)	0.426	n.s.	n.s.	n.s.
Age (years, mean ± SD)	42.2 ± 19.2 (*n *= 402)	40.9 ± 19.8 (*n *= 79)	42.5 ± 18.6 (*n *= 118)	42.9 ± 19.4 (*n *= 147)	0.787	n.s.	n.s.	n.s.
Male patients (%)	63.7 (*n *= 256)	65.8 (*n *= 52)	58.5 (*n *= 69)	64.6 (*n *= 95)	0.481	n.s.	n.s.	n.s.
ISS (points, mean ± SD)	25.9 ± 11.4 (*n *= 402)	21.3 ± 9.0 (*n *= 79)	27.6 ± 11.2 (*n *= 118)	29.6 ± 10.9 (*n *= 147)	<0.001	<0.001	n.s.	<0.001
ISS ≥16 (%)	85.6 (*n *= 344)	74.7 (*n *= 59)	90.8 (*n *= 107)	97.4 (*n *= 143)	<0.001	<0.001	0.030	0.005
AIS head ≥3 (%)	23.3 (*n *= 344)	30.4 (*n *= 24)	26.3 (*n *= 31)	17.0 (*n *= 25)	0.048	0.027	0.071	0.627
AIS thorax ≥3 (%)	54.4 (*n *= 344)	50.6 (*n *= 40)	59.3 (*n *= 70)	52.4 (*n *= 77)	0.397	n.s.	n.s.	n.s.
AIS abdomen ≥3 (%)	25.6 (*n *= 344)	15.2 (*n *= 12)	25.4 (*n *= 30)	31.3 (*n *= 46)	0.030	0.010	0.339	0.110
AIS extremities ≥3 (%)	83.1 (*n *= 344)	58.2 (*n *= 46)	85.6 (*n *= 101)	100.0 (*n *= 147)	<0.001	<0.001	0.019	<0.001
New-ISS (points, mean ± SD)	29.5 ± 11.1 (*n *= 402)	24.9 ± 9.1 (*n *= 79)	30.9 ± 10.3 (*n *= 118)	33.3 ± 11.3 (*n *= 118)	<0.001	<0.001	n.s.	<0.001
PTS (points, mean ± SD)	32.4 ± 17.0 (*n *= 402)	24.5 ± 14.9 (*n *= 79)	33.4 ± 16.2 (*n *= 118)	36.8 ± 17.7 (*n *= 147)	<0.001	<0.001	n.s.	<0.001
GCS on scene (points, mean ± SD)	11.5 ± 4.5 (*n *= 284)	12.3 ± 3.9 (*n *= 64)	11.6 ± 4.0 (*n *= 77)	11.9 ± 4.2 (*n *= 104)	0.625	n.s.	n.s.	n.s.
Time from accident to hospital admission (minutes, mean ± SD)	73.0 ± 36.1 (*n *= 210)	73.7 ± 40.7 (*n *= 41)	73. 9 ± 38.1 (*n *= 57)	68.2 ± 29.5 (*n *= 92)	0.714	n.s.	n.s.	n.s.
Time in the trauma room (minutes, mean ± SD)	72.4 ± 40.8 (*n *= 353)	78.4 ± 43.6 (*n *= 70)	74.8 ± 45.1 (*n *= 98)	68.0 ± 36.0 (*n *= 133)	0.301	n.s.	n.s.	n.s.

AIS, Abbreviated Injury Scale; GCS, Glasgow Coma Scale; ISS, Injury Severity Score; n.s., not significant; PTS, Hannover Polytrauma Score.

### Parameters of the initial resuscitation period

The mean time period from accident to hospital arrival was 73 minutes, whereas the time from the trauma-room admission to the ICU or the operating theatre had a mean 72 minutes. Table 2 shows the preclinical vital signs as well as the administered fluid volume in relation to the pelvic-ring fractures (according to the Tile/OTA classification). In the prehospital phase, both patients with mechanically unstable pelvic-ring fracture types B and C showed significantly worse vital signs compared with type A, demonstrating a higher ratio of patients in shock already in the field for both of them. This higher physiological instability was related to administration of significantly higher volumes of crystalloids and colloids in fracture types B and C.

**Table 2:** Vital parameters, infusion volume, and blood transfusions during the initial resuscitation period (prehospital phase and time from arrival in the emergency department until arrival on ICU) in relation to the fracture type of the pelvic ring according Tile/OTA fracture classification

			Type of pelvic ring fracture according Tile/OTA fracture classification				Pair-wise comparison		
	
		Total	Type A	Type B	Type C	Overall test	*P *value (A vs. C)	*P *value (B vs. C)	*P *value (A vs. B)
Prehospital phase	Systolic blood pressure (mm Hg, mean ± SD)	109 ± 31 (*n *= 227)	120 ± 26 (*n *= 59)	113 ± 31 (*n *= 73)	100 ± 32 (*n *= 95)	<0.001	<0.001	0.005	n.s.
	Ratio of patients in shock in the field (systolic blood pressure <90 mm Hg) (%)	19.4 (*n *= 227)	11.9 (*n *= 7)	16.4 (*n *= 12)	26.3 (*n *= 25)	0.065	n.s.	n.s.	n.s.
	Heart rate (beats/min., mean ± SD)	99 ± 23 (*n *= 231)	91 ± 18 (*n *= 62)	101 ± 20 (*n *= 71)	102 ± 27 (*n *= 98)	0.006	0.003	n.s.	0.006
	Oxygen saturation (%, mean ± SD)	93 ± 10 (*n *= 230)	94 ± 6 (*n *= 53)	92 ± 8 (*n *= 65)	91 ± 13 (*n *= 81)	0.578	n.s.	n.s.	n.s.
	Infusion volume (crystalloids + colloids) (ml, mean ± SD)	1,464 ± 1,032 (*n *= 258)	1,072 ± 881 (*n *= 67)	1,608 ± 1,096 (*n *= 79)	1,596 ± 1,017 (*n *= 112)	<0.001	< 0.001	0.953	<0.001

Emergency department (ED)	Systolic blood pressure on ED arrival (mm Hg, mean ± SD)	115 ± 27 (*n *= 304)	123 ± 25 (*n *= 69)	115 ± 23 (*n *= 103)	111 ± 31 (*n *= 132)	0.013	0.004	n.s.	n.s.
	Ratio of patients in shock on ED arrival (systolic blood pressure <90 mm Hg) (%)	12.2 (*n *= 304)	4.3 (*n *= 3)	8.7 (*n *= 9)	18.9 (*n *= 25)	0.005	0.005	0.039	0.366
	Heart rate (beats/min., mean ± SD)	91 ± 21 (*n *= 297)	87 ± 19 (*n *= 68)	92 ± 20 (*n *= 100)	93 ± 22 (*n *= 129)	0.143	n.s.	n.s.	n.s.
	Infusion volume ED to ICU (crystalloids + colloids) (ml, mean ± SD)	2,884 ± 2,471 (*n *= 290)	1,991 ± 1,975 (*n *= 67)	2,645 ± 2,438 (*n *= 103)	3,587 ± 2,565 (*n *= 120)	<0.001	< 0.001	< 0.001	0.118
	Packed red blood cell concentrates ED to ICU (PRBC) (units, mean ± SD)	3.4 ± 7.2 (*n *= 344)	2.1 ± 5.7 (*n *= 42)	3.0 ± 6.2 (*n *= 54)	4.5 ± 8.5 (*n *= 83)	<0.001	< 0.001	n.s.	0.005
	Fresh frozen plasma ED to ICU (FFP) (units, mean ± SD)	3.0 ± 6.6 (*n *= 344)	1.7 ± 4.9 (*n *= 37)	2.7 ± 6.3 (*n *= 52)	3.8 ± 7.5 (*n *= 73)	0.010	0.003	n.s.	n.s.

Total initial resuscitation period	Total infusion volume during the initial resuscitation period (crystalloids + colloids) (ml, mean ± SD)	4,626 ± 3,079 (*n *= 222)	3,173 ± 2,613 (*n *= 57)	4,677 ± 2,976 (*n *= 72)	5,476 ± 3,121 (*n *= 93)	<0.001	< 0.001	0.061	<0.001

n.s., not significant.

On arrival in the ED, the formerly worse vital signs appear to have improved, but with increasingly pelvic ring instability, fluids and transfusions further increased significantly over time. The latter is, however, limited to the type C fractures. Finally, the total volume of crystalloids and colloids administered during whole Initial Resuscitation Period was higher in types B and C.

### ICU parameters

The overall rate of MODS was 25.8%, and 5.2% of the investigated patients developed a sepsis. In total, all patients were ventilated for 6.4 ± 10.1 days, and the average ICU length of stay was 11.5 ± 11.8 days. With increasingly pelvic-ring instability, the ratio of MODS, as well as the duration of ventilation and of stay in the ICU increased significantly, particularly in the presence of type C fractures. Notably, these findings disagreed for patients with sepsis (Table 3).

**Table 3:** ICU parameters (ICU length of stay, ventilation length of time, rate of multiple-organ-dysfunction syndrome, and sepsis) in relation to the fracture type of the pelvic ring according to the Tile/OTA classification

		Type of pelvic-ring fracture according to Tile/OTA fracture classification				Pair-wise comparison		
			
	Total	Type A	Type B	Type C	Overall test	*P *value (A vs. C)	*P *value (B vs. C)	*P *value (A vs. B)
Multiple-organ-dysfunction syndrome (%)	25.8 (*n *= 326)	22.1 (*n *= 17)	19.6 (*n *= 22)	32.9 (*n *= 45)	0.042	n.s.	0.014	n.s.
Sepsis (%)	5.2 (*n *= 325)	3.9 (*n *= 3)	2.7 (*n *= 3)	8.1 (*n *= 11)	0.137	n.s.	n.s.	n.s.
Days on ventilation (days, mean ± SD)	6.4 ± 10.1 (*n *= 344)	4.3 ± 6.7 (*n *= 79)	6.0 ± 10.4 (*n *= 118)	7.7 ± 11.2 (*n *= 147)	0.039	0.016	n.s.	n.s.
ICU length of stay (days, mean ± SD)	11.5 ± 11.8 (*n *= 344)	9.4 ± 9.9 (*n *= 79)	10.6 ± 11.2 (*n *= 118)	13.3 ± 12.9 (*n *= 147)	0.031	0.016	n.s.	n.s.

n.s., not significant.

## Discussion

By matching for the first time the German Pelvic Injury Register with the TraumaRegister DGU, statements are feasible about the recent practice of initial fluid management for different Tile/OTA types of pelvic-ring fractures as well as about the patient's posttraumatic course, including ICU data. Because of the innovative idea of interrelating two trauma registers, a stand-alone technical-notes manuscript with detailed descriptions of the matching process and its weakness in method will be published separately. Nevertheless, the literature contains a multitude of studies about the management of fluid resuscitation of trauma patients, and even a Cochrane review exists [21]. Some studies focus on the prehospital setting [23,29-31], some on the emergency department [3,5,13,17,20], and some on the time in the operating theatre [22]. Starting with the innovative resuscitation study of Bickell *et al*. [29] from 1994, their "immediate resuscitation group" received means of 870 ± 667 ml Ringers acetate before arrival at the hospital and 1,608 ± 1,201 ml in the ED. Today, 15 years later, the standard regimen in fluid resuscitation in our study has doubled [29]. Certainly, we must keep in mind that Bickell *et al*. investigated penetrating torso injuries and not multiple-trauma patients with pelvic disruptions. Looking for similar populations, Gruen *et al*. [13] studied 312 patients with pelvic-ring fractures, type B/C with shock, receiving 5,900 ± 4,000 ml of crystalloids given in the ED. This amount of fluid is even more than we saw in the selective group of type C fractures (3,587 ± 2,565 ml). In contrast, the ratio of our patients in shock on hospital admission was only about 20%, explaining the less fluid given by our study. More recent studies described the administration of 4,271 ± 2,428 ml and 2,750 ml of fluids given in the ED. Thereby, Verbeek *et al*. [17] also analyzed hemodynamically unstable pelvic fractures, and Giannoudis *et al*. [3] even represented the median value of the nonsurviving group [3,17]. In 2011, Hussmann *et al*. [23] showed that increasingly, preclinical volume led to a slight elevation of lethality as well as of transfused packed red blood cells concentrates (PRBCs) in multiply injured patients after severe abdominal and pelvic trauma, and recommended for both a moderate prehospital volume replacement.

In summary, despite the well-known problem of comparability of all studies in the field of multiple-trauma patients, the large fluid volumes of our study confirm the actuality of traditional pelvis-specific trauma algorithms [14,18]. Low-volume resuscitation seems not yet to be accepted in practice in managing this special patient entity. Regarding the bleeding risk for different Tile/OTA types of pelvic-ring fractures, our fluid volumes in the prehospital phase suggest that type B and C fractures are different from type A fractures, whereas in the hospital setting type A and B are rather similar and type C is different. A possible explanation could be the volume triggered and synchronized changes of the shock ratio with no significant differences in the pre-hospital phase that turns after ED admission into significant more type C fractures with haemorrhagic shock. Notably, the type C group was related to the presence of a higher percentage of severe concomitant injuries (AIS ≥3) that can contribute to increased hemorrhage (that is, abdomen and extremities). Conversely, the hemorrhagic shock cannot be entirely attributed to the pelvic fracture alone, as has been stated by other authors [8-10,25,32,33]. A potential bias in treatment might exist once the diagnosis is known, as type C fractures are considered a more severe injury, and the patient receives more fluids than the fluids being administered in response to resuscitation. Nevertheless, bleeding and resuscitation reflect a dynamic process. Looking at the total initial resuscitation period, the infusion volume as well as the transfused PRBCs of the type B and C fractures are different from type A fractures. In accord with others, in our study, increased pelvic-ring instability resulted in increased injury severity (that is, ISS, New-ISS, and PTS), and thus elevated mortality [2,5]. The highest mortality in the group of the complex pelvic injuries can be explained by its dominance of type B/C fractures, with significantly higher overall trauma load and injury severity [1,2,8,10]. Unquestionably, the pelvic fracture is only one variable among many (that is, age, shock, head injury, abdominal or chest injury, and extremity injury, which contribute to mortality risk [8-10,25,34,35]). Regarding the ICU data, the overall rates of 5.2% of sepsis and 25.8% of MODS we found are quite similar to those reported in the literature [2,20,23,32]. The same is true for the ventilated days and the length of ICU stay; both variants are shorter compared with those reported in older literature [2,13,17,19,23]. Analogous to the mortality, the outstanding position of the type C fractures in sepsis, ventilation, and ICU stay is beyond doubt influenced by the concomitant abdominal and extremity injuries with more pronounced shock in the initial resuscitation phase, as well as the higher overall trauma load and injury severity compared with the other subtypes [8-10,25,32,33]. Notably, multiple-trauma patients are at high risk for developing MODS and/or sepsis [34-36]. This also applies for the type A fractures that appeared in about three fourths of patients with an ISS ≥16. The higher rate of sepsis and MODS of type A in comparison to type B fractures might be the consequence of the exclusion of the prognostic unfavorable AIS head > 4 population before starting the analysis meanwhile, almost all of the AIS head > 4 population had type B/C fractures. No doubt certain limitations exist in our study. First, weakness is found in the method of interrelating two trauma registers. In-depth analysis of that will be presented in the future in a separate article. In brief, in the technical notes manuscript, the degree of data validation of twofold documented records (that is, ISS, systolic blood pressure, and hemoglobin on ED arrival, as well as mortality will be analyzed). Second, the matching of PIR and TR data resulted in a respectable study population of 402 multiple-trauma patients with pelvic disruptions. The majority of the hospitals that contributed to the two data registers were Level I trauma centers with additional specialization in pelvic trauma. Thus, the experience in managing pelvic injuries is biased and might explain the low mortality rate of 7.5% compared with that in the literature [5,7,9,17,23]. Another point is the incompleteness of the assessed parameters reflected by the differing numbers of patients for different parameters. Although data-entry errors rates for unknown or missing information are familiar problems in medical registries, the error rates in our study did not differ from the range of 19% to 76% out of the literature [37].

## Conclusions

The present study confirms the actuality of traditional trauma algorithms with initial massive fluid resuscitation in the recent therapy of multiple-trauma patients with pelvic disruptions. Low-volume resuscitation seems not yet accepted in practice in managing this special patient entity. Mechanically unstable pelvic-ring fractures type B/C (according to Tile/OTA classification) form a distinct entity that must be considered in future trauma algorithms.

## Key messages

• Massive fluid resuscitation in the initial resuscitation period still reflects recent practice in multiple-trauma patients with pelvic disruptions.

• Low-volume resuscitation seems not yet accepted in practice in managing this special patient entity.

• Increased pelvic-ring instability was related to increased fluid/transfusion requirements in the initial resuscitation period, as well as higher-severity injury score, the presence of shock and complications, and higher mortality rate.

• Mechanically unstable pelvic-ring fractures type B/C, according Tile/OTA classification, should be considered in future trauma algorithms.

## Abbreviations

AIS: Abbreviated Injury Scale; ATLS: Advanced Trauma Life Support; DGU: Deutsche Gesellschaft für Unfallchirurgie (transl. German Society for Trauma Surgery); ED: emergency department; FFP: fresh frozen plasma; GCS: Glasgow Coma Scale; ICU: intensive care unit; ISS: Injury Severity Score; MODS: multiple-organ-dysfunction syndrome; n.s.: not significant; OTA: Orthopaedic Trauma Association; PIR: (German) Pelvic Injury Register; PRBC; packed red blood cell concentrate; PTS: Hannover Polytrauma Score; SD: standard deviation; SOFA: Sequential Organ Failure Assessment; TR: TraumaRegister DGU.

## Competing interests

T. Paffrath is member of the steering committee of the Traumaregister DGU. UC and T Pohlemann are members of the steering committee of the German Pelvic Injury Register of the German Trauma Society. All authors declare that they have no competing interests, nor were this study or the authors funded.

## Authors' contributions

MB, UN, and TPA had full access to all of the data in the study and take responsibility for the integrity of the data and the accuracy of the data analysis. Study concept and design: SF, TPA, and MB. Acquisition of data: AP, MM, UC, BB, SF, and TPO. Analysis and interpretation of data: UN, TPA, and MB. Drafting the manuscript: UN, TPA, and MB. Critical revision of the manuscript for important intellectual content: AP, MM, UC, BB, SF, and TPO. Statistical analysis: UN. All authors read and approved the manuscript.
